# CGRP‐Targeted Therapy in Vestibular Migraine—How Strong Is the Evidence?

**DOI:** 10.1111/ene.70688

**Published:** 2026-07-03

**Authors:** Alexander Andrea Tarnutzer, Patricia Castro Abarca, Diego Kaski

**Affiliations:** ^1^ Neurology, Cantonal Hospital of Baden Baden Switzerland; ^2^ Faculty of Medicine University of Zurich Zurich Switzerland; ^3^ School of Allied Health Sciences, Faculty of Health and Life Sciences De Montfort University Leicester UK; ^4^ SENSE Research Unit, Department of Clinical and Movement Neurosciences UCL London UK

**Keywords:** calcitonin‐gene related peptide (CGRP), monoclonal antibodies, primary headache, small molecules, systematic review, vestibular migraine

## Abstract

**Background:**

Medications for migraine prevention targeting the calcitonin gene‐related peptide (CGRP) pathway have substantially reduced symptom burden in many patients that have failed previous treatment strategies. In contrast, data on treatment response to monoclonal antibodies (mAbs) or small molecule receptor antagonists (gepants) in patients suffering from vestibular migraine (VM) is scarce and preliminary.

**Methods:**

We discuss the existing literature on VM‐prevention using mAbs and gepants with a special focus on biases and limitations such as small sample sizes, retrospective study design, lack of blinding, and patient selection.

**Results:**

Studies identified (*n* = 8) assessed different mAbs and gepants and generally reported improvement of vestibular symptoms and scores used, but were often of small sample size and lacked blinding and control groups. In a single randomized controlled trial, a significant treatment response to galcanezumab was identified, with a medium to large effect size (ranging between 0.56 and 1.02) for dizzy days reported and on scores applied (dizziness handicap inventory [DHI] and Vestibular Migraine Patient Assessment Tool and Handicap Inventory [VM‐PATHI]).

**Conclusions:**

Data on anti‐CGRP treatments for VM remain limited, and efficacy established in headache migraine cannot be straightforwardly extrapolated to VM given differences in underlying pathophysiology. There remains a risk that initial effect estimates may diminish over time, with early outcomes partially inflated by a novelty effect, expectancy, and more nuanced methodological approaches. Targeted treatment options for this common and often debilitating condition are genuinely welcomed, but the current evidence warrants careful interpretation for CGRP‐related therapies.

## 1 Introduction

Targeting the calcitonin gene‐related peptide (CGRP) pathway by use of monoclonal antibodies (mAbs) or small molecule receptor antagonists (gepants) has offered an exciting and targeted therapeutic option for migraine prevention. Monoclonal antibodies against CGRP or its receptor, as well as gepants, have demonstrated efficacy in both acute and preventive migraine therapy [1]. This development has encouraged the use of these new, though expensive drugs. In a 2024 position statement, the American Headache Society elevated CGRP‐targeted therapies to first‐line status in migraine prevention [2]. In many other countries and healthcare systems, regulatory frameworks and prescribing criteria have been introduced to restrict the use of anti‐CGRP therapies in migraine treatment owing to their cost. The use of these drugs has instead been reserved for those with frequent and severe migraine headache attacks and lacking benefit from established drugs, either due to poor treatment response, side effects or contraindications for use.

Vestibular migraine (VM) is a neurological condition that causes episodic vestibular symptoms associated with other features of migraine [3], and has a lifetime prevalence of 1.0%–2.7%. Treatment strategies for VM have mostly mirrored those of migraine headaches [4, 5]. In one systematic review, five randomized controlled trials (RCTs) published between 2014 and 2024 (including 419 patients) comparing six treatments (propranolol, flunarizine, venlafaxine, valproic acid, galcanezumab, *Lactobacillus casei* Shirota) were included in a network meta‐analysis. All treatments significantly reduced monthly vertigo attacks versus controls/placebo [4]. In a Cochrane analysis performed in 2023, the authors concluded that “there is very limited evidence from placebo‐controlled randomized trials regarding the efficacy and potential harms of pharmacological interventions for prophylaxis of vestibular migraine” [6]. Such conclusions are not unexpected from a systematic review of novel treatments for an episodic neurological disorder. But as we sit in this unprecedented era of having a new medication that seems to explicitly target mechanisms that underpin cephalgic migraine, it seems prudent to question whether there is sufficient evidence to promote the use of anti‐CGRP‐related treatment in VM specifically.

The recent network meta‐analysis from Vasireddy and colleagues on this topic included only 17 patients treated with galcanezumab, suggesting that data is sparse. Our review of the literature (Pubmed/Embase) focusing on original publications reporting on treatment responses to anti‐CGRP‐monoclonal antibodies or gepants in VM‐patients (performed on March 9th 2026) identified 195 unique citations, with eight manuscripts (and 212 patients; Table 1).

**TABLE 1:** Summary of studies exploring the use of anti‐CGRP therapies in patients with vestibular migraine.

Citation	Study design	Total subjects	Medication used (in *n* patients)	Primary outcome	Blinding/randomization	Patient selection	Research support by Pharma	Main results
Hoskin et al., 2021 [7]	Retrospective	28 (25 analyzed)	Erenumab (*n* = 11), Galcanezumab (*n* = 6), Fremanezumab (*n* = 9), Ubrogepant (*n* = 2)	Patient‐reported improvement	N/A (Retrospective)	VM patients who were prescribed CGRP after failure with other conventional migraine medication.	No	84% showed some degree of improvement and 60% demonstrated a moderate–significant improvement.
Inui T et al., 2025 [8]	Case report	1	Erenumab (*n* = 1)	Headaches and dizziness perception	N/A (Case report)	VM patient with concomitant MD with previous unsuccessful treatment.	No	Reduction in headaches and dizziness plus improvement in, DHI (86%), HIT‐6 (84%) and MIDAS (36%).
Kouga et al., 2024 [9]	Retrospective	23 (including 12 controls)	Erenumab (*n* = 2), Galcanezumab (*n* = 10)	DHI score	N/A (Retrospective)/Not randomized (retrospective controls)	VM patients treated with anti‐CGRP mAbs (CGRP group) after failure with other coventional migraine medication, and 11 patients receiving standard care (control group).	No	Significant reductions in DHI (54%) plus reduction in the vertigo/dizziness attacks per month (61%), VAS (64%), and HIT‐6 (27%) scores in the CGRP group compared to controls.
Lovato et al., 2023 [10]	Retrospective	23	Erenumab (*n* = 23)	Migraine days over the last 3 months.	N/A (Retrospective)	VM patients who were prescribed CGRP after documented unsuccessful attempts with > 3 migraine preventive medication.	No	A 59% reduction in migraine days during last 3 months and a 73% reduction in DHI score after treatment was observed.
Russo CV et al., 2023 [11]	Prospective observational	50	Fremanezumab (*n* = 25), Galcanezumab (*n* = 18), Erenumab (*n* = 7)	Monthly days with vertigo/dizziness.	No (Observational)	VM patients who were prescribed CGRP with history of 3 failed treatments with validated migraine preventatives at standard dose for at least 2 months.	No directly, but some authors declare receiving financial compensation from pharmaceutical companies.	90% of patients had ≥ 50% vertigo days reduction and 80% of patients had a MIDAS reduction of at least 50%.
Sharon JD et al., 2024 [12]	Double‐blind RCT	40 (including 22 controls)	Galcanezumab (*n* = 18)	VM‐PATHI score	Double‐blind/Yes—randomization (galcanezumab vs. placebo)	VM or probable VM plus > 4 definite dizzy days during baseline, VM‐PATHI score > 25	Yes, Eli Lilly and Company	VM‐PATHI reduction of 31.3% in treatment group versus 11.2% in control group. DHI and DDD reduction of 35.5% and 63.1% respectively in treatment group compared to 14.6% and 31.1% in control group.
Shao et al., 2025 [13]	Prospective self‐controlled	33	Rimegepant (*n* = 33)	Vestibular symptom days	No (Observational)	VM or probable VM	No	The days with vestibular sx had a 52% reduction from baseline, while DHI and HIT‐6 had a 58% and 18% reduction respectively.
Patel et al., 2026 [14]	Retrospective	17*	Rimegepant (*n* = 15), Ubrogepant (*n* = 10), Atogepant (*n* = 4), Zavegepant (*n* = 1)	Self‐perceived improvement	N/A (Retrospective)	VM or probable VM who were treated with a small molecule CGRP antagonist.	No	76% of patients declare relief of their most bothersome symptom and 84% of patients claimed medications helped to manage their sx.

Abbreviations: CGRP, calcitonin gene‐related peptide; DDD, definite dizzy days; DHI, dizziness handicap inventory; HIT‐6, headache impact test; MD, Menière's disease; MIDAS, Migraine disability assessment; N/A, not available; RCT, randomized controlled trial; sx, symptoms; VM, vestibular migraine; VM‐PATHI, Vestibular Migraine Patient Assessment Tool and Handicap Inventory.

* Note that some patients used several gepants in combination for both prevention and acute treatment. Thus, the total number of substances used is larger than the patient sample size.

The literature is limited and heterogeneous, comprising a single case study [8], four retrospective case series [7, 9, 10, 14], two prospective observational studies [11, 13] and one randomized controlled trial [12]. Patient selection varies: several studies included only individuals with treatment‐refractory disease, while others did not report prior treatment response, limiting comparability and generalizability.

Across studies, 127 patients received monoclonal antibodies, 52 gepants, and 33 placebo. Outcomes were primarily patient‐reported, including migraine days, dizzy days, and Dizziness Handicap Inventory (DHI) scores. Although improvements were consistently reported, effect sizes varied widely and reliance on subjective measures without standardization constrains interpretation. The sole randomized controlled trial demonstrated significantly greater improvement with galcanezumab versus placebo across multiple endpoints, including dizzy days and DHI, with relatively large effect sizes. However, manufacturer support introduces potential bias. Overall, current evidence suggests possible benefit but is constrained by small sample sizes, heterogeneity, and methodological limitations, underscoring the need for larger, independent trials.

Overall, we argue that the current body of evidence for use of anti‐CGRP‐related treatment in VM remains preliminary. The trial design of most included studies lacks rigor and does not meet standards of high‐quality treatment trials. Methodological limitations identified include lack of blinding, lack of control groups, and highly selective patient cohorts. In fact, only a single RCT reported superior benefits of galcanezumab over placebo in reducing dizzy days per month in patients with VM or probable VM [12].

Several challenges must therefore be addressed before anti‐CGRP therapies can be recommended for widespread use in patients with VM, including uncertainties about true efficacy and the magnitude of the placebo response. The placebo effect may be amplified by the way these drugs are presented to patients, particularly when promoted as “new” or “more effective” treatments. In addition, the absence of blinding and control groups in many studies likely inflates reported benefits (i.e., overestimation of treatment response). Apparent efficacy may diminish over time as treatment is offered to a broader and less selectively defined VM population, and as higher‐quality trials incorporating adequate blinding and placebo controls are conducted.

Another important challenge relates to possible differences in pathological mechanisms underlying migraine headache and VM. Differences in response to triptan therapy for acute attacks highlight potential pathophysiological differences between these two entities. In the acute treatment of migraine headaches, pain freedom is achieved 37%–50% of the time [15]. Instead, a recent double‐blinded RCT has demonstrated no benefit for rizatriptan for vestibular symptoms in VM [16]. While the pathophysiology of VM is incompletely understood, neuroimaging studies support the presence of structural and functional abnormalities within vestibulo–thalamo–cortical networks in affected patients [17]. By contrast, alternative hypotheses propose a peripheral mechanism, whereby transient inner ear hypoperfusion during migrainous attacks, potentially due to vasospasm, gives rise to vertiginous symptoms [5] and the role of CGRP in these is less clearly established than for the pain pathway for migraine [1]. This emphasizes the need for VM‐specific treatment protocols and high‐quality RCTs that are independent of pharmaceutical support.

If robust VM‐specific trials demonstrate meaningful benefit, national prescribing and reimbursement criteria for anti‐CGRP therapies will need to be reconsidered. Current eligibility frameworks are largely based on headache frequency and severity, reflecting the migraine evidence base rather than VM itself. Consequently, patients with a predominantly vestibular phenotype, who often report less prominent headaches, may not meet existing thresholds despite significant disability. Any revision of access criteria would require not only clinical validation but also health‐economic evaluation to ensure equitable and cost‐effective implementation. We argue that the evidence supporting the use of CGRP‐targeted therapy in vestibular migraine is not sufficiently strong to recommend this as a first‐line treatment.

## Author Contributions

**Alexander Andrea Tarnutzer:** conceptualization, investigation, methodology, validation, data curation, formal analysis, writing – original draft, writing – review and editing. **Patricia Castro Abarca:** conceptualization, writing – original draft, writing – review and editing, validation, methodology, data curation, formal analysis. **Diego Kaski:** conceptualization, writing – original draft, writing – review and editing, validation, methodology, formal analysis, data curation, supervision.

## Conflicts of Interest

The authors declare no conflicts of interest.

## Data Availability

The data that support the findings of this study are available on request from the corresponding author. The data are not publicly available due to privacy or ethical restrictions.
